# TNF-α inhibits SCF, ghrelin, and substance P expressions through the NF-κB pathway activation in interstitial cells of Cajal

**DOI:** 10.1590/1414-431X20187065

**Published:** 2018-04-23

**Authors:** Keyu Ren, Chunming Yong, Hao Yuan, Bin Cao, Kun Zhao, Jin Wang

**Affiliations:** 1 Department of Gastroenterology, The Affiliated Hospital of Qingdao University, Qingdao, China; 2 Department of Emergency Medicine, The Affiliated Hospital of Qingdao University, Qingdao, China; 3 Department of Pathology, School of Basic Medicine, Medical College of Qingdao University, Qingdao, Shandong, China

**Keywords:** TNF-α, SCF, Ghrelin, Substance P, NF-κB, Interstitial cells of Cajal

## Abstract

Ulcerative colitis is a chronic inflammatory disease of the colon where intestinal motility is disturbed. Interstitial cells of Cajal (ICC) are required to maintain normal intestinal motility. In the present study, we assessed the effect of tumor necrosis factor-alpha (TNF-α) on viability and apoptosis of ICC, as well as on the expression of stem cell factor (SCF), ghrelin, and substance P. ICC were derived from the small intestines of Swiss albino mice. Cell viability and apoptosis were measured using CCK-8 assay and flow cytometry, respectively. ELISA was used to measure the concentrations of IL-1β, IL-6, ghrelin, substance P, and endothelin-1. Quantitative RT-PCR was used to measure the expression of SCF. Western blotting was used to measure the expression of apoptosis-related proteins, interleukins, SCF, and NF-κB signaling pathway proteins. TNF-α induced inflammatory injury in ICC by decreasing cell viability and increasing apoptosis and levels of IL-1β and IL-6. TNF-α decreased the levels of SCF, ghrelin, and substance P, but had no effect on endothelin-1. TNF-α down-regulated expressions of SCF, ghrelin, and substance P by activating the NF-κB pathway in ICC. In conclusion, TNF-α down-regulated the expressions of SCF, ghrelin, and substance P via the activation of the NF-κB pathway in ICC.

## Introduction

Ulcerative colitis is an idiopathic, chronic inflammatory disease of the colon, most commonly affecting people aged 30 to 40 years (1,2). The pathogenesis is multifactorial, involving dysregulated immune responses, genetic predisposition, epithelial barrier defects, and environmental factors (3). Ulcerative colitis is characterized by mucosal inflammation of the rectum and colon. Major symptoms include bloody diarrhea, fatigue, abdominal cramps, urgency, and fever (4–6). Ulcerative colitis is most prevalent in Europe (505 per 100.000 persons), followed by Canada (248 per 100.000 persons), and the USA (214 per 100.000 persons). The primary aim of medical management is to induce and maintain clinical and endoscopic remission (7). Treatments of ulcerative colitis include aminosalicylates for mild to moderate disease, topical and systemic steroids for disease flares, and immunosuppressants and biological drugs for moderate to severe disease. Colectomy is required for patients with refractory disease or colonic neoplasia (3); however, effective control of ulcerative colitis still remains difficult.

Currently, studies on the pathogenesis of ulcerative colitis are mostly focused on immunity, heredity, infection, and intestinal flora factors (8–10); very few studies have focused on the changes of intestinal motility (11). Interstitial cells of Cajal (ICC), expression of stem cell factor (SCF), ghrelin, and endothelin-1 play important roles in intestinal motility (12–14). ICC are mesenchymal cells that are distributed along the gastrointestinal (GI) tract and function as pacemaker or neuromediator cells between nerves and smooth muscle cells. ICC are needed for normal intestinal motility (15). ICC express the receptor tyrosine kinase, c-Kit, which is a recognized marker for ICC (16). It has been reported that the ultrastructure of ICC is disturbed in patients with ulcerative colitis (17).

The c-Kit/SCF signal is responsible for phenotypic maintenance, proliferation, and differentiation (18). SCF is decreased in mice models of ulcerative colitis, indicating that the pathogenesis of ulcerative colitis is related to c-Kit/SCF (19). Ghrelin is a gut-brain peptide that exerts endocrine effects (food intake control) and plays important roles in energy homeostasis and modulation of immune and inflammatory responses (20). Jung et al. (21) proposed that circulating ghrelin levels and obestatin/ghrelin ratio served as markers of activity in ulcerative colitis patients. Endothelins are peptides that exert various biological effects, including vasoconstriction and stimulation of cell proliferation (22). Murch et al. (12) reported high endothelin-1 immunoreactivity in Crohn's disease and ulcerative colitis. Thus, it is confirmed that ICC, SCF, ghrelin, and endothelin-1 are associated with ulcerative colitis.

Cytokines, such as tumor necrosis factor-alpha (TNF-α) and interleukin (IL), play important roles in the inflammatory process of ulcerative colitis. Increasing evidence indicates a genetic association between TNF-α and ulcerative colitis. Additionally, increased TNF-α levels have been noted in patients with ulcerative colitis. TNF-α is an important constituent in the pathophysiology of ulcerative colitis, and thus, agents targeting TNF-α have been studied for ulcerative colitis (23). In the present study, we aimed to assess the effects of TNF-α on components related to intestinal motility using ICC, derived from mice.

## Material and Methods

### Cell culture and treatment

Animal care and studies were done according to the guidelines of the ethics committee of Qingdao University. Six Swiss albino mice (20–25 g) were used in this study. The small intestines of the mice were removed (from 1 cm below the pyloric ring to the cecum) and opened along the mesenteric border. Luminal contents were washed by Krebs-Ringer bicarbonate solution, and the tissues were pinned to the base of a Sylgard dish. Mucosa was removed by sharp dissection. Small tissue strips of intestinal muscle (consisting of both circular and longitudinal muscles) were equilibrated in nominally Ca^2+^-free solution (5.36 mM KCl, 125 mM NaCl, 0.34 mM NaOH, 0.44 mM NaHCO_3_, 10 mM glucose, 2.9 mM sucrose, and 11 mM HEPES; pH 7.4) for 30 min. The cells were then dispersed in a solution containing collagenase (1.3 mg/mL; Worthington Biochemical, USA), bovine serum albumin (BSA; 2 mg/mL; Sigma-Aldrich, USA), trypsin inhibitor (Sigma-Aldrich, 2 mg/mL), and ATP magnesium salt (0.27 mg/mL). The cells were placed onto sterile glass cover slips coated with murine collagen (2.5 μg/mL; Falcon/BD, USA) in a 35-mm culture dish. The cells were then cultured at 37°C in an incubator with 95% O_2_-5% CO_2_ atmosphere in smooth muscle growth medium (SMGM; Clonetics, USA) supplemented with 2% antibiotics/antimycotics (Gibco, USA), and murine SCF (5 ng/mL; Sigma-Aldrich). ICC were identified using an immunological technique by anti-c-Kit antibody (Santa Cruz, USA) at a dilution of 1:50 for 20 min. ICC were morphologically different from other cell types in the culture, and were identified using phase contrast microscopy after verification with anti-c-Kit antibody. The cells were treated with different concentrations (10 to 40 ng/mL) of TNF-α. SN50 (20 µM) was used as nuclear factor-kappaB (NF-κB) inhibitor.

### Cell counting kit-8 (CCK-8) assay

Cell viability was assessed using CCK-8 assay (Dojindo Molecular Technologies, USA). Cells were seeded on a 96-well plate, with 5000 cells/well. After stimulation, CCK-8 solution was added to the culture medium, and the cultures were incubated for 1 h at 37°C in humidified 95% air and 5% CO_2_. Absorbance was measured at 450 nm using Microplate Reader (Bio-Rad, USA).

### Apoptosis assay

Flow cytometry analysis was done to identify and quantify apoptotic cells using Annexin V-FITC/PI apoptosis detection kit (Beijing Biosea Biotechnology, China). Cells (100,000 cells/well) were seeded in a 6-well plate. Treated cells were washed twice with cold phosphate buffered saline (PBS) and resuspended in buffer. The adherent and floating cells were combined and treated according to the manufacturer's instructions and measured with flow cytometer (Beckman Coulter, USA) to differentiate apoptotic cells (Annexin-V positive and PI-negative) from necrotic cells (Annexin-V and PI-positive).

### Enzyme-linked immunosorbent assay (ELISA)

Cells (100,000 cells/well) were plated onto 96-well plates and cultured at 37°C overnight. Then, after desired treatments, culture supernatant was collected for measurements using ELISA kits, according to the recommended protocols. Levels of ghrelin and endothelin-1 were assessed by kits from Abcam (UK). Level of substance P was determined by kit from Cayman Chemical (USA). Concentrations of inflammatory cytokines were measured by kits from R&D Systems (UK).

### Quantitative reverse transcription polymerase chain reaction (qRT-PCR)

Total RNA was isolated from transfected cells using Trizol reagent (Invitrogen, USA) and treated with DNaseI (Promega, USA). Reverse transcription was performed using MultiScribe Reverse Transcriptase (Thermo Fischer Scientific, USA) and random hexamers or oligo(dT). The reverse transcription conditions were 10 min at 25°C, 30 min at 48°C, and a final step of 5 min at 95°C. Real-time PCR for evaluation of SCF mRNA level was performed using Powerup SYBR Green Master Mix (Thermo Fischer Scientific) following the protocol of supplier. Fold relative expression was calculated according to the 2^-ΔΔCt^ method (24), normalizing to GAPDH.

### Western blotting

The proteins used for western blotting were extracted using RIPA lysis buffer (Beyotime Biotechnology, China) supplemented with protease inhibitors (Roche, USA). The proteins were quantified using BCA™ Protein Assay Kit (Pierce, USA). The western blotting system was established using Bio-Rad Bis-Tris Gel system according to the manufacturer's instructions. Proteins in the gels were transferred to polyvinylidene difluoride (PVDF) membranes, followed by blocking with 5% non-fat milk. Primary antibodies, such as anti-Bcl-2 (ab196495), anti-Bax (ab182733), anti-pro caspase-3 (ab115183), anti-cleaved caspase-3 (ab49822), anti-IL-1β (ab200478), anti-IL-6 (ab208113), anti-SCF (ab64677), anti-p65 (ab16502), anti-phospho (p)-p65 (ab86299), IκBα, inhibitor of nuclear factor κB α (IκBα; ab32518), p-IκBα (ab133462), GAPDH (ab181603, all Abcam), anti-pro caspase-9 (9504), and anti-cleaved caspase-9 (9509; both Cell Signaling Technology, USA) antibodies were incubated with the membrane at 4°C overnight, followed by washing and incubation with secondary antibody marked by horseradish peroxidase (ab205718, Abcam) for 1 h at room temperature. After rinsing, the polyvinylidene difluoride membrane-carried blots and antibodies were transferred into Bio-Rad ChemiDoc™ XRS system, and then 200 μL Immobilon Western Chemiluminescent HRP substrate (Millipore, USA) was added to cover the membrane surface. The signals were captured using Image Lab™ software (Bio-Rad).

### Statistical analysis

All experiments were repeated three times. The results of multiple experiments are reported as means±SD. Statistical analyses were performed using SPSS 19.0 statistical software (USA). P values for comparisons between two groups were calculated using unpaired two-tailed *t*-test, and for three groups or more were calculated using one-way analysis of variance (ANOVA) with Holm-Sidak's *post hoc* test. P<0.05 was considered to indicate a statistically significant result.

## Results

### TNF-α induced inflammatory injury in ICC

**Figure 1.**
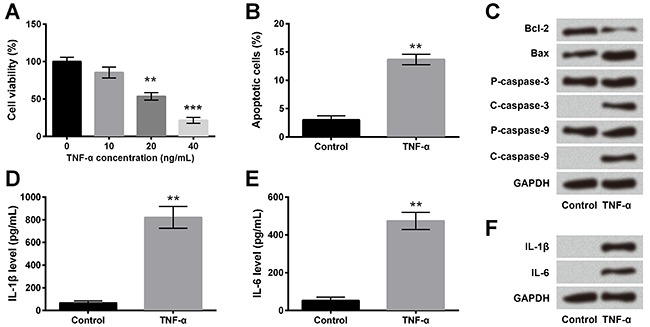
TNF-α induced inflammatory injury in interstitial cells of Cajal (ICC). ICC were treated with TNF-α at 10, 20, or 40 ng/mL concentrations. *A*, Cell viability was measured using CCK-8 assay. *B*, Apoptosis was measured using flow cytometry assay. *C*, Expression of apoptosis-associated proteins was determined by western blotting. *D-F*, Concentrations of IL-1β and IL-6 were measured using ELISA and western blotting. Data are reported as means±SD. **P<0.01, ***P<0.001, compared to control (*t*-test and ANOVA). CCK-8: cell counting kit-8; ELISA: enzyme-linked immunosorbant assay; GAPDH: glyceraldehyde 3-phosphate dehydrogenase; IL: interleukin; TNF-α: tumor necrosis factor-alpha; P-: pro; C-: cleaved.

ICC were treated with TNF-α at 10, 20, or 40 ng/mL concentrations, and then cell viability was measured using CCK-8 assay and apoptosis was determined using flow cytometry. As shown in Figure 1A, TNF-α significantly decreased cell viability at 20 ng/mL (F(3,8)=38.82, P<0.01) and 40 ng/mL concentrations (F(3,8)=38.82, P<0.001). The concentration of TNF-α for subsequent experiments was 20 ng/mL. TNF-α significantly increased apoptosis compared to the control (P<0.01; Figure 1B). Western blotting analysis also confirmed this finding, where TNF-α decreased the expression of anti-apoptotic protein (Bcl-2) and increased the expressions of pro-apoptotic proteins (Bax and cleaved caspases 3 and 9) compared to the control (Figure 1C). Then, we measured the concentrations of pro-inflammatory cytokines, IL-1β and IL-6, using ELISA. As shown in Figure 1D and E, TNF-α increased the levels of IL-1β and IL-6 compared to the control (both P<0.01). Western blotting confirmed these findings as TNF-α increased the expressions of IL-1β and IL-6 (Figure 1F). These findings indicated that TNF-α induced inflammatory injury in ICC by decreasing cell viability and increasing apoptosis and secretion of pro-inflammatory cytokines.

### TNF-α decreased the levels of SCF, ghrelin, and substance P

**Figure 2.**
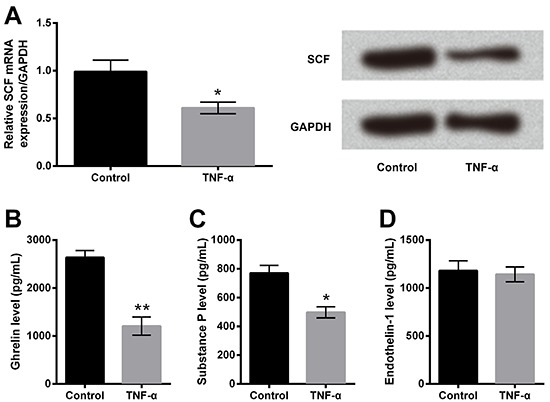
TNF-α decreased the levels of stem cell factor (SCF), ghrelin, and substance P. Interstitial cells of Cajal (ICC) were treated with 20 ng/mL TNF-α and non-treated cells were used as control. *A*, qRT-PCR and western blotting were used to measure the expression of SCF. ELISA was used to measure the concentrations of (*B*) ghrelin, (*C*) substance P, and (*D*) endothelin-1. Data are reported as means±SD. *P<0.05, **P<0.01, compared to control (*t*-test). ELISA: enzyme-linked immunosorbent assay; GAPDH: glyceraldehyde 3-phosphate dehydrogenase; qRT-PCR: quantitative reverse transcription polymerase chain reaction; TNF-α: tumor necrosis factor-alpha.

Next, we assessed the effect of TNF-α on SCF using qRT-PCR and western blotting analyses. Results showed that TNF-α significantly decreased the expression of SCF compared to the control (P<0.05; Figure 2A). Then, we assessed the effect of TNF-α on the levels of ghrelin, substance P, and endothelin-1 using ELISA, and found that TNF-α significantly deceased the levels of ghrelin (P<0.01; Figure 2B) and substance P (P<0.05; Figure 2C), but had no effect on endothelin-1 level (Figure 2D). These findings indicated that TNF-α down-regulated expressions of SCF, ghrelin, and substance P in ICC.

### TNF-α inhibited the expression of SCF, ghrelin, and substance P by activating the NF-κB pathway

**Figure 3.**
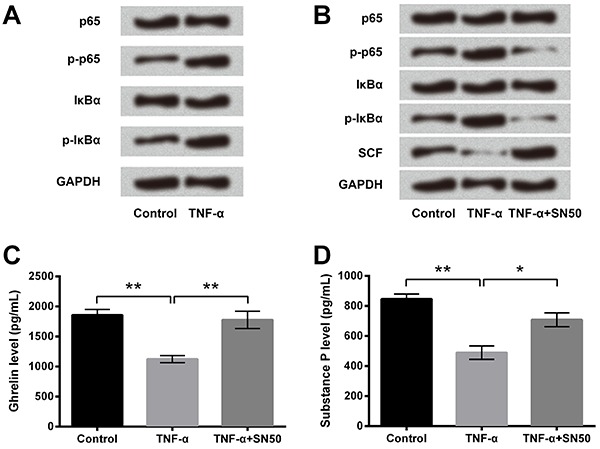
TNF-α inhibited the expressions of stem cell factor (SCF), ghrelin, and substance P by activating the NF-κB pathway. Interstitial cells of Cajal (ICC) were treated with 20 ng/mL TNF-α and non-treated cells were used as control. *A* and *B*, Expressions of SCF and NF-κB signaling pathway proteins were measured by western blotting. ELISA was used to assess the effect of TNF-α and TNF-α+SN50 on the levels of (*C*) ghrelin and (*D*) substance P. Data are reported as means±SD. *P<0.05, **P<0.01 (ANOVA). ELISA: enzyme-linked immunosorbent assay; GAPDH: glyceraldehyde 3-phosphate dehydrogenase; NF-κB: nuclear factor kappa B; TNF-α: tumor necrosis factor-alpha.

Lastly, the effect of TNF-α on the NF-κB signaling pathway proteins (p65 and IκBα) was examined using western blotting. As shown in Figure 3A, TNF-α increased the phosphorylation of p65 and IκBα compared to the control. However, addition of NF-κB inhibitor (SN50, 20 µM) reversed these effects by decreasing the phosphorylation of the NF-κB pathway proteins and increasing the expression of SCF (Figure 3B). Then, ELISA was done to assess the effects of TNF-α and TNF-α+SN50 on the levels of ghrelin and substance P. Results showed that addition of SN50 reversed the effects of TNF-α by increasing the levels of ghrelin (F(2,6)=14.72, P<0.01, Figure 3C) and substance P (F (2,6)=18.26, P<0.05, Figure 3D), compared to the TNF-α group. These findings indicated that TNF-α inhibits the expression of SCF, ghrelin, and substance P by activating the NF-κB signaling pathway.

## Discussion

In the present study, we assessed the effects of TNF-α on viability and apoptosis of ICC, and on the levels of IL-1β, IL-6, SCF, ghrelin, endothelin-1, and substance P in ICC. We also examined the involvement of the NF-κB signaling pathway in the effects of TNF-α on expression of SCF, ghrelin, and substance P. Results revealed that TNF-α decreased ICC viability, increased apoptosis, increased IL-1β and IL-6 levels, and decreased SCF, ghrelin, and substance P levels by activating the NF-κB signaling pathway.

TNF-α, lipopolysaccharide, and toll-like receptor 4 are crucial in inducing phenotypic changes in ICC under an inflammatory microenvironment in the gut (25). Eisenman et al. (26) suggested that TNF-α, which was secreted from M1 macrophages, could induce c-Kit loss and ICC injury through caspase-dependent apoptosis *in vitro*. In a mouse model of ulcerative colitis, which was induced by dextran sulfate sodium, expressions of TNF-α, IL-1β, and IL-6 were markedly up-regulated in the colon, resulting in intestinal mucosal inflammation (27). A previous study also reported that IL-6 release in inflammatory microenvironment could down-regulate c-Kit expression and decrease ICC activities (28). In our study, TNF-α decreased ICC viability and increased apoptosis partially through caspase-dependent pathway, along with increases of IL-1β and IL-6 levels. Taken together, these findings indicate that TNF-α adversely affects ICC in ulcerative colitis.

In a rat model of inflammatory bowel diseases, bone marrow mesenchymal stromal cells and soluble SCF played a synergistic role in mucosal cell regeneration following experimentally induced intestinal injury (29). Thus, administration of SCF may be of therapeutic value in inflammatory bowel diseases, including ulcerative colitis. SCF is also considered a ligand of c-Kit, and the activation of SCF/Kit pathway is essential for development and maintenance of ICC networks (30). In our study, TNF-α significantly down-regulated the expression of SCF at mRNA and protein levels in ICC compared to the control, which is in agreement with a study by Rusten et al. (31), showing that TNF-α inhibits SCF-induced proliferation of human bone marrow progenitor cells *in vitro*. Our study also proved that TNF-α-induced down-regulation of SCF could be reversed by inhibition of the NF-κB pathway in ICC. Similarly, a study by Jin et al. (32) illustrated that curcumin up-regulated SCF expression through inactivating the NF-κB pathway.

The majority of circulating levels of ghrelin is produced in the stomach. Ghrelin exerts a range of immunological effects. For example, it decreases leptin-induced pro-inflammatory responses and inhibits secretion of TNF-α, IL-1β, IL-6, and IL-8. Due to the functional and anatomical link of ghrelin with inflammation and the GI tract, ghrelin has been studied in a variety of GI disorders, including colitis (33). Maduzia et al. (34) found that acetic acid-induced colitis was effectively ameliorated by administration of ghrelin, which was related to the anti-inflammatory effects of ghrelin. Another study also reported that experimental colitis could be attenuated by ghrelin via repressing the NF-κB pathway in mice (35). In agreement with those findings, we found that TNF-α inhibited the expression of ghrelin by activating the NF-κB pathway.

Substance P is a neurotransmitter found in colonic mucosa, which can alter gut vascular, immunologic, and motor phenomena. Thus, it may have a vital role in the pathogenesis of ulcerative colitis. Szitter et al. (36) summarized that, in inflamed human gut, the alterations of substance P expression are contradictory. Other studies also reported the dual role of substance P in colitis development. On the one hand, substance P could induce inflammatory response via evoking activation of the NF-κB signaling pathway (37). On the other hand, substance P promotes proliferation and mobilizes stem cells to the site of injury, leading to facilitation of mucosal healing in colitis (38). Hong et al. (39) also reported that dextran sulfate sodium-induced intestinal damage was effectively ameliorated by substance P via enrichment of M2 macrophages and regulatory T cells. In our study, we found that the expression of substance P was down-regulated by TNF-α by activating the NF-κB pathway in ICC.

In conclusion, TNF-α induced inflammatory injury in ICC by decreasing cell viability and increasing apoptosis and levels of IL-1β and IL-6. We also found that TNF-α inhibited the expression of SCF, ghrelin, and substance P by activating the NF-κB signaling pathway. Our study provides a regulatory mechanism of TNF-α in ICC and emphasizes the importance of the NF-κB signaling pathway in development of ulcerative colitis. The inhibition of the NF-κB signaling pathway may be a possible therapeutic strategy for treatment of ulcerative colitis; however, additional clinical experiments should be done in the future.
